# A Case of Exertional Heat Stroke Complicated by Hypoxic Hepatitis

**DOI:** 10.1155/2020/8724285

**Published:** 2020-03-30

**Authors:** Bertram K. Woitok, Shawki Bahmad, Gregor Lindner

**Affiliations:** Department of General Internal and Emergency Medicine, Buergerspital Solothurn, Solothurn, Switzerland

## Abstract

**Background:**

Exertional heat stroke is a life-threatening condition often complicated by multiorgan failure. We hereby present a case of a 25-year-old male presenting with syncope after a 10  km run in 28°C outside temperature who developed acute liver failure. *Case Presentation*. Initial temperature was found to be 41.1°C, and cooling measures were rapidly applied. He suffered from acute renal failure and rhabdomyolysis and proceeded to acute liver failure (ASAT 6100 U/l and ALAT 6561 U/l) due to hypoxic hepatitis on day 3. He did not meet criteria for emergency liver transplantation and recovered on supportive care.

**Conclusions:**

Acute liver failure due to heat stroke is a life-threatening condition with often delayed onset, which nevertheless resolves on supportive care in the majority of cases; thus, a delayed referral to transplant seems to be reasonable.

## 1. Background

Exertional heat stroke is a life-threatening condition caused by excess heat generated from muscular exercise that exceeds the body's ability to dissipate it at the same rate. It is a form of hyperthermia associated with a systemic inflammatory response leading to a syndrome of multiorgan dysfunction in which encephalopathy predominates. Typical organ dysfunctions consists of liver failure due to hypoxic hepatitis, acute kidney injury, rhabdomyolysis, and disseminated intravascular coagulation [1, 2].

## 2. Case Presentation

We present case of severe heat stroke complicated by acute liver failure and rhabdomyolysis in a 25-year-old male after running 10  km in 28°C outside temperature presenting with syncope upon finishing the run. Paramedics on-site reported an initial GCS of 3, a body temperature of 41.1°C, and a blood glucose of 3.3  mmol/l. Cool packs were placed inguinal and 20  g parenteral glucose were administered.

The patient reported nausea and vomiting as well as a severe headache. His prior medical history was significant for an autism spectrum disorder, and otherwise unremarkable, he was taking no medication. The vital signs upon arrival showed GCS 15 with a tympanic temperature of 38.6°C, blood pressure was normal at 129/98  mmHg, and heart rate was 130  min^−1^. The patient was hyperventilating at a respiratory rate of 45  min^−1^ and peripheral oxygen saturation was 98% breathing ambient air. The physical examination was otherwise unremarkable.

Laboratory findings are presented in Table 1. A urinalysis showed a leukocyturia and haematuria, and drug screen was negative. Leukocyte and erythrocyte casts as well as bacteria were present. An electrocardiogram and X-ray of the chest showed normal results.

After rehydrating with Ringers lactate solution, the body temperature stayed high between 38 and 39°C. He received symptomatic therapy with ondansetron. As leucocyte casts were present in urine, we treated a possible urinary tract infection with ceftriaxon, and to counteract possible concomitant fever, paracetamol and also metamizole were administered. The patient was admitted to the general medicine ward for further observation and rehydration.

On the third day after admission, the patient showed a slightly better general condition. Laboratory examinations revealed acute liver injury with ASAT 6100 U/l and ALAT 6561 U/l. The CK level elevated to a peak of 15969 U/l with a slightly improved kidney function (Table 1). The virus serology for hepatitis B, C, and E as well as HIV was negative.

As our patient did not meet the Kings College criteria for emergency liver transplantation, he was not referred to a tertiary centre for emergent transplant listing. Liver and kidney injury resolved with aggressive fluid resuscitation. The patient was discharged in good condition at the 8th day.

## 3. Discussion

Heat stroke is a form of hyperthermia associated with a systemic inflammatory response leading to a syndrome of multiorgan dysfunction in which encephalopathy predominates. Encephalopathy may be subtle, manifesting only as inappropriate behavior or impaired judgment. Heat stress leads to cutaneous vasodilation and splanchnic vasoconstriction, and also it triggers an acute phase response. The acute phase response plays a significant role in the development of hepatic injury, as is shown by an increase of interleukin-6 levels [1]. In addition, numerous animal models show that blockage of the release pathways for the cytokines IL-1*β*, IL-6, TNF-*α*, CD11b, CD68, I*κ*B*α*, and NF-*κ*B p65 and microRNA-155 [3–5].

Successful treatments with emergency liver transplantation [6–15], MARS [16], plasma exchange alone [17, 18], and hemofiltration [19] as well as cold dialysis and hemofiltration [20] have been published. There is growing evidence that conservative management seems to be sufficient for the management of most cases, and a delayed transplant strategy is reasonable [8, 12, 21–31], with cases reporting successful transplant after delaying transplantation in favour of spontaneous recovery [8, 12]. Most reported cases survived, but fatalities have been reported due to liver failure [8, 32–34] or transplant complications [35–37]. Nevertheless, early induction of cooling treatment, ideally reaching <38.9°C within 30 minutes, seems to be paramount [20, 38, 39]. Also, the administration of N-acetyl-cysteine was shown to improve transplant-free survival [40, 41] and has been reported in acute liver failure secondary to heat stroke [8, 21, 22]. It could also be shown that hypophosphatemia <0.5 mmol/l at admission, as was present in our patient, could predict the occurrence of acute liver failure [42], although it is unclear whether there is a causative connection. Predictors of an unfavorable outcome are core temperature >42°C, rapid multiorgan failure requiring artificial organ support and need of vasopressors [8].

## 4. Conclusions

We here present a case of heat stroke complicated by hypoxic hepatitis, acute renal failure, and rhabdomyolysis. A possible predisposing factor was urinary tract infection. Rapid cooling was achieved through convection cooling and transfer to air conditioned environment. Estimating the grade of encephalopathy was difficult due to autism spectrum disorder and mild hypoglycemia, but we interpreted the syncope as well as nausea and severe headache as manifestation of heat induced encephalopathy. Of note was the delayed onset of liver injury, presenting on day three after admission, which was also reported by the majority of published cases. After reviewing the literature, we decided to observe for spontaneous recovery and delay referral to a tertiary centre which was ultimately not necessary due to recovery.

The weakness of our case is that not all possible etiologies for acute liver injury were excluded as ceruloplasmin, iron studies, and an autoimmune panel were not obtained. In addition, there were no coagulation studies at the time of liver injury and the patient had received a single dose of paracetamol. We still decided to present the case due to the suggestive history and spontaneous resolve of the liver injury, which would neither be expected in metabolic or autoimmune liver injury or following a single dose of paracetamol, to raise awareness to this possible life-threatening complication of heat stroke.

## Figures and Tables

**Table 1:** Blood test results, patient's evolution.

Test	Normal range	D1	D3	D4	D6
Hemoglobin (g/l)	137–165	182	156		154
WBC (10^9^/l)	3.9–9.5	18.8	14.1		9.1
Platelet count (10^9^/l)	140–360	90	97		132
INR		1.11			1.16
Sodium (mmol/l)	136–145	145	137	138	138
Potassium (mmol/l)	3.6–5.1	4.1	4.4	4.2	4.1
Calcium (mmol/l)	2.10–2.55	2.80		2.07	
Phosphorous (mmol/l)	0.74–1.52	0.22		0.61	
Creatinine (µmol/l)	64–104	195	127	103	103
Urea (mmol/l)	3.2–7.4	9.7		5.3	
CK (U/l)	30–200	842	15969	8743	2308
Total bilirubin (*μ*mol/l)	<21	16	30	56	39
Aspartate aminotransferase (U/l)	5–35	48	6100	3062	411
Alanine aminotransferase (U/l)	<45	26	6561	5745	2811
Alkaline phosphatase (U/L)	40–150		72	82	86
Gamma-glutamyl transferase (U/L)	12–64	21	33	106	150
Lipase (U/l)	8–78	55			19
C-reactive protein (mg/l)	<5.1	0.3	2.4		1.1
Lactate (mmol/l)	0.5–2.2	4.7			

WBC: white blood cells; CK: creatinine phosphokinase; INR: international normalized ratio.

## Abbreviations

ASAT: Aspartate aminotransferase
ALAT: Alanine aminotransferase
CK: Creatinine phosphokinase.
